# Changes in Breast Cancer Presentation during COVID-19: Experience in an Urban Academic Center

**DOI:** 10.1155/2023/6278236

**Published:** 2023-06-09

**Authors:** Brian Diskin, Nakisa Pourkey, Freya Schnabel, Pabel Miah, Charles DiMaggio, Deborah Axelrod, Richard Shapiro, Amber A. Guth

**Affiliations:** ^1^Division of Breast Surgery, Department of Surgery, NYU Langone Health, 550 First Avenue, New York, NY 10016, USA; ^2^Department of Surgery, New York University School of Medicine, 550 First Avenue, New York, NY 10016, USA

## Abstract

The COVID-19 pandemic strained healthcare systems worldwide, delaying breast cancer screening and surgery. In 2019, approximately 80% of breast cancers in the U.S. were diagnosed on screening examinations, with 76.4% of eligible Medicare patients undergoing screening at least every two years. Since the start of the pandemic, many women have been reluctant to seek elective screening mammography, even with the lifting of pandemic-related restrictions in access to routine healthcare. We describe the effect of the COVID-19 pandemic on breast cancer presentation at a tertiary academic medical center greatly impacted by the pandemic.

## 1. Introduction

Breast cancer is the most common cancer among women and results in approximately 15% of cancer deaths in developed countries [1]. In 2013, over 700,000 person years of life was lost due to breast cancer, and the estimated diagnosis for new breast cancer in 2017 was over 250,000 in the United States. With the advent of new screening techniques, current guidelines for early detection reduce breast cancer-related deaths by 20-40% [2].

Although there are various resources regarding recommended breast cancer screening such as the NCCN and the USPSTF, providers use guidelines and tailor to patients based on an individuals' risk factors [3]. In recent years, there has been a decrease in mortality and invasive breast surgeries have decreased—a direct result of improved screening [4, 5]. In 2019, approximately 80% of breast cancers in the U.S. were diagnosed on screening examinations, with 76.4% of eligible Medicare patients undergoing screening at least every two years. As a result of early detection and advances in treatment methods such as combining breast conservation and selective lymph node procedures with appropriate systemic and radiation treatment, mortality has been significantly reduced [3].

Coronavirus disease 2019 (COVID-19) was first identified in Wuhan, China, in December 2019, and the ensuing pandemic created a public health emergency, which resulted in the delay of delivery of healthcare worldwide [6]. Worldwide routine cancer screening and treatment were adversely affected [7]. The first case of COVID-19 was identified in New York City on March 1, 2020. On March 23, 2020, Governor Andrew Cuomo of New York State issued an executive order, directing the reallocation of healthcare resources towards treating patients with COVID-19 [7]. Elective surgeries were suspended in New York City, and drastic lockdown measures were employed. Intensive care units (ICU) replaced conventional hospital wards, and medical personnel—regardless of specialty training—were reassigned to the care of COVID-19 patients. Outpatient visits and routine cancer screenings were delayed, and telemedicine was expanded [8]. Out of fear for contracting the virus, patients were reluctant to comply with their recommended screening [9]. Rates of cancer screening in the United States sharply declined in March through May 2020, with an absolute deficit for breast cancer screening estimated to be 3.9 million. Modeling the effects of COVID-19 on cancer screening and treatment for breast cancer estimated a 1% increase in deaths [10].

Due to the pandemic, routine screening exams were suspended, and several countries initiated modified guidelines for breast cancer during the pandemic. Of note, Canada suggested delaying evaluation for 4 weeks for annual surveillance of breast cancer survivors, cyclical breast pain, low suspicion lesions in elderly patients, BI-RADS 3–4A lesions, young patients with likely fibroadenoma, males with likely gynecomastia, and proven cysts [11]. Even with suggested precautions, delay in following prepandemic standard of care screening recommendations likely diminished.

The aim of this study was to determine how the COVID-19 pandemic and the ensuing lockdowns, reallocation of healthcare resources, and delays in breast cancer screening and treatment altered the presentation and oncologic treatment of breast cancer at our urban academic medical center in NYC, the epicenter of the early phase of the pandemic in the US [12].

## 2. Methods

Our institutional IRB-approved breast cancer database was queried for patients enrolled during two time periods, comparing patients undergoing a first surgical procedure before the start of the pandemic (4/1/2019-3/31/2020) to those treated during the year after the pandemic started (4/1/2020-3/31/2021). 349 patients were in the 2019 cohort (prepandemic), and 246 were in the 2020 cohort (postpandemic). It was noted that elective cancer surgery was paused for a 3-week period, ending on 4/20/2020, and then limited availability until 6/1/2022. Variables of interest included age, race, method of detection, palpability, histologic subtype and staging, cancer specific treatments, and radiation uptake.

## 3. Results

**Figure 1:**
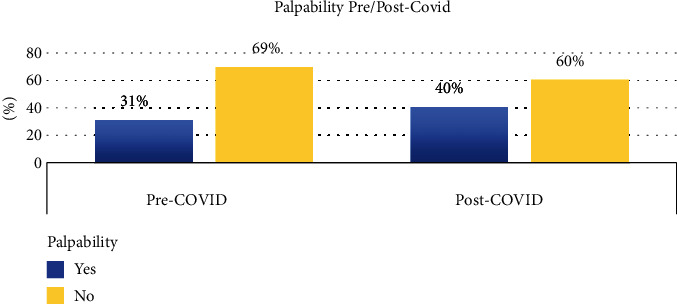
Palpable breast disease pre-/post-COVID-19.

Baseline characteristics were similar between the two groups. There were no differences in age, race, prior biopsy history, medical history, lifestyle factors, risk factors, family history, age at presentation, nodal status, or operation type (Table 1). We found that fewer cancers were detected on routine mammography post-COVID-19 vs. pre-COVID-19. We found a significant increase in detection of breast cancer through self-exams in 2020 compared to 2019 (Figure 1). There was a trend toward decreases in breast lesions detected through provider physical exams. Palpability on presentation also significantly increased. The rate of invasive ductal cancers increased within the 2020 cohort, and the rate of detection dropped for DCIS and for invasive lobular cancers (ILC) Table 1. More patients were treated with neoadjuvant systemic therapy, and 36 of 45 eligible early-stage breast cancer patients accepted neoadjuvant hormonal therapy during the period that elective cancer surgery was on hold [13]. Patients received radiation therapy less frequently during the pandemic.

## 4. Discussion

Breast cancer makes up 24% of all cancer diagnoses and is a significant public health issue worldwide [1]. Early diagnosis is critical to long-term survival. The COVID-19 pandemic caused a significant delay in healthcare delivery, including oncologic screening and treatment. All aspects of breast cancer care were affected, and the presentation of advanced tumors—including inflammatory breast tumors—was reported worldwide [14]. Others have found that breast cancer patients treated during the first year of the COVID-19 pandemic presented with more advanced, larger tumors, and an increased incidence increased axillary involvement, likely secondary to a sharp decrease in the level of mammographic screening [6, 15]. For example, one study from Taiwan found that breast cancer screening decreased by 22% [16]. Other studies have found even steeper decreases in levels of screening, with data from Indiana revealing a 34% decrease in screening mammography [6]. Many patients cancelled their screening mammography appointments secondary to fear of contracting the coronavirus [8]. In our study, we sought to characterize the effect of the COVID-19 pandemic on the presentation and treatment of breast cancer at our urban academic medical center.

The virus spread rapidly worldwide after being first identified on China in December 2019. The first case of COVID-19 was identified on March 1, 2020, in New York City, which quickly became an epicenter of the pandemic. By mid-April 2020, over 100,000 cases had been reported, along with approximately 13,000 deaths [17]. Given the severity with which the COVID-19 pandemic affected New York City, we hypothesized that the negative effects on breast cancer screening and treatment would be especially pronounced in our institution.

Our data demonstrated that patients at our academic medical center at New York City presented with more palpable and invasive breast cancers during the COVID-19 pandemic compared to the preceding year, and fewer patients with DCIS and invasive lobular cancers typically detected following screening mammography. While stage migration with significant increases in diagnosis of late-stage cancers has been described, in our population, the stage shift occurred in early-stage breast cancer, with decreases in DCIS and increases in stages I-II, with the higher stages III-IV essentially unchanged. This reflects the effect of delay in our previously highly screened population, with an average screening delay of more than 3 months, and many patients missing their yearly screening altogether.

While many interactions during COVID-19 were via telemedicine, radiation therapy requires daily office visits, and patient fear of exposure contributed to the lower rate of radiation. It is clear that screening reduces the incidence of higher-stage disease. In the 400,000 women cohort from Italy, comparing attenders' and nonattenders' stage-specific breast cancer incidence, the authors estimated that screening attendance is associated with a reduction of nearly 30% for stages II+ [18]. Given the increase in invasiveness and stage of breast cancers diagnosed during the COVID-19 pandemic, this study emphasizes the importance of screening for the diagnosis and treatment of breast cancer, even in the face of a concurrent health crisis.

One drawback with our study is that the database enrolls surgical patients only: thus, the patients who presented during this period with recognized stage IV disease were not captured.

In addition, although surrounding institutions halted elective surgeries for several months, the institution used for this study had a brief pause for only 3 week. Long-term follow-up will be critical to determine the effect of the pandemic on breast cancer survival.

## 5. Conclusion

Given the increase in invasiveness and stage of breast cancers diagnosed during the COVID-19 pandemic, this study emphasizes the importance of screening for the diagnosis and treatment of breast cancer.

## Figures and Tables

**Table 1:** Patient population before COVID-19 (2019) and after COVID-19 (2020).

Variable	2019 population (*N* = 349)	2020 population (*N* = 246)	*P* value
Method of detection			
Self-exam	19.80%	26.0%	0.0688
Mammography	67.0%	60.0%	
Palpability	31.50%	39.20%	0.0533
Neoadjuvant therapy	8.30%	10.20%	0.4384
Radiation therapy	65.0%	54%	<0.0001
Age at presentation	60.04	60.68	0.6171
Type of surgery			
Breast conserving surgery	69%	66%	<0.8508
Mastectomy	31%	34%	
Histology			
IDC	60.70%	66.7%	0.5822
DCIS	20.9%	16.7%	
ILC	10.6%	8.10%	

## Data Availability

The patient data used to support the findings of this study are included within the article.
